# Health-related quality of life after posterior vertebral column resection in children: comparison with healthy controls

**DOI:** 10.1007/s00590-021-03064-3

**Published:** 2021-06-24

**Authors:** Johanna Syvänen, Linda Helenius, Arimatias Raitio, Paul Gerdhem, Elias Diarbakerli, Ilkka Helenius

**Affiliations:** 1 Departments of Pediatric Orthopedic Surgery and Anaesthesia and Intensive Care, University of Turku, Turku University Hospital, Kiinanmyllynkatu 4–8, 20521 Turku, Finland; 2 Department of Clinical Science, Intervention and Technology (CLINTEC), Karolinska Institutet, Stockholm, Sweden; 3 Department of Reconstructive Orthopaedics, Karolinska University Hospital, Stockholm, Sweden; 4 Department of Orthopaedics and Traumatology, University of Helsinki, Helsinki University Hospital, Helsinki, Finland

**Keywords:** Vertebral column resection, Scoliosis, Kyphosis, Health-related quality of life

## Abstract

**Purpose:**

Vertebral column resection (VCR) is a technique performed for short, angular spinal deformities. Several studies have reported good radiographic results with VCR regarding curve correction. However, only a few studies have reported the impact of this technique on the health-related quality-of-life measures (HRQoL).

**Methods:**

A single surgeon series of 27 consecutive children (mean age at surgery 12.3 years, range 1.1–20.7 years) undergoing posterior VCR with a minimum of 2-year follow-up. The comparison was made to age- and gender-matched healthy controls. Outcome measures included Scoliosis Research Society (SRS) questionnaire both pre- and postoperatively, radiographic outcomes, and complications.

**Results:**

The average major curve correction was 60.3% in the VCR patients. Complications were noted in 12 out of 27 (44%) of the VCR patients but all patients recovered fully during follow-up. The SRS pain domain scores improved significantly after VCR (*p* = 0.0002). The SRS total and domain scores were significantly lower than in the healthy controls especially in the self-image and function domains, but the pain and activity domains improved from preoperative to similar level than in the control group.

**Conclusions:**

HRQoL showed significant improvement in pain scores despite 44% risk of transient complications after VCR in pediatric patients. This health-related quality-of-life improvement remained at a significantly lower level than in the healthy control group.

**Level of Evidence:**

Therapeutic Level III.

## Introduction

Vertebral column resection (VCR) is defined as a resection of the dorsal components and at least one vertebral body with the caudal and cranial intervertebral disks. Typical indications in the pediatric age group include short angular and/or severe spinal deformities [1, 2]. An untreated severe scoliosis sometimes needs apical VCR to allow adequate correction [1, 3]. VCR was originally performed using an anteroposterior approach [4], but more recently it has been performed using a posterior only approach (PVCR) [1, 5, 6].

Several studies have reported good radiographic results, but the risk of spinal cord and/or neural element deficits appears to be higher than in typical pedicle screw instrumentation [1, 3, 5–7]. Lenke et al. published the first series of PVCR to pediatric patients and reported no spinal cord-related complications [1]. Curve correction of approximately 60% can be achieved with PVCR [3, 8].

To the best of our knowledge, data on the health-related quality of life (HRQoL) after PVCR in pediatric patients are limited [3, 8] and no comparisons of HRQoL at the end of follow-up to healthy controls have been published. In the series of Helenius et al., most patients reported high satisfaction in Scoliosis Research Society (SRS)-24 scores [3]. One long-term study reporting HRQoL measures in a combined series of pediatric and adult patients exists [8].

We aimed to compare HRQoL in pediatric patients with congenital or idiopathic scoliosis undergoing PVCR with healthy controls. We hypothesized that PVCR would improve HRQoL, but the quality of life would still remain at lower level than in healthy controls.

## Methods

This was a retrospective study using a prospectively collected data on consecutive children undergoing PVCR by a single orthopedic spine surgeon. All consecutive PVCR procedures from January 2007 to January 2018 were evaluated for eligibility. Exclusion criteria included associated neurological or syndromic condition preventing assessment of HRQoL. Results were compared with age and sex matched healthy controls. Age matching was performed based on the age at final follow-up.

Twenty-seven otherwise healthy children (mean age 12.3 years, range 1.1 to 20.5) with minimum 2 years follow-up underwent PVCR for short angular congenital scoliosis or kyphosis or severe scoliosis (from January 2007 to January 2018 (mean follow-up 4.0 years, range 2 to 11 years). The indications included congenital kyphosis (*n* = 7), congenital scoliosis (*n* = 6), congenital kyphoscoliosis (*n* = 8), global kyphosis (*n* = 2), metatropic dysplasia and thoracic kyphosis (*n* = 1) and congenital dislocation of vertebral column (*n* = 1). This cohort did not include single posterolateral hemivertebra resections. The study received approval from the Ethics Committee of the Hospital District (Tables 1 and 2).

**Table 1:** Preoperative and postoperative patient data

Variable	VCR patients (*N* = 27)	Healthy controls (*N* = 54)
Mean age at surgery	12.3 (1.1–20.7)	
Mean age at final follow-up	15.3 (5.7–25.7)	17.1 (9.0–30.6)
*Sex*		
Male	17	34
Female	10	20
*Preoperative neurological deficit*		
No	26	
Yes	2	
*Revision surgery*		
No	26	
Yes	2

**Table 2:** Patient characteristics and operation details

Patient/gender	Age at the operation (yrs)	Diagnosis	Follow-up (months)	Level of VCR	Fused levels	Prior surgery	Estimated blood loss (mL)	Operation time (h)
1/M	12.6	CS, CK, Jeune syndrome	115	T8 + T9	T4–L3	–	3200	5.17
2/F	4.7	CS	115	T4	T3–T5	–	200	3.08
3/M	10.3	CS, CK	113	T12	T8–L3	–	750	5.08
4/F	15.0	CS, CK	107	T12 + L1 partial	T9–L4	–	1400	6.17
5/F	14.4	CK	104	T11	T8–L2	–	900	5.33
6/F	18.3	CK	103	L1	T6–L4	–	680	5.25
7/M	17.3	CK	99	T11	T8–L2	–	700	5
8/F	20.7	Metatropic dysplasia, thoracal kyphosis	89	T8	T3–T12	–	1150	5.08
9/M	15.5	CS, CK	95	T12	T9–L3	–	1380	5.25
10/F	12.6	CS, CK	91	T10	T7–L1	–	700	5
11/M	1.1	Congenital luxation of vertebrae T11/L1	91	T12	T10–L2	–	120	4.17
12/M	11.9	Juvenile scoliosis	87	T7	**T1–L3**	–	2020	5.5
13/M	14.5	CS	77	T11	T9–L1	–	260	3.25
14/F	14.5	CS	70	L4	L2–S1	–	580	5.0
15/F	11.7	CK	65	T8	T5–T11	–	860	4.83
16/F	8.3	CS	60	S1	L5–S2	–	560	4
17/M	14.2	CS, CK	59	T7	T1–T10	–	560	4.41
18/M	16.8	CS, CK	54	T11	T8–L2	–	635	3.75
19/M	14.6	CK	34	T11	T7–L2	–	660	3.41
20/M	9.1	CS, CK	27	T4	T1–L2	+	1280	4.75
21/M	8.1	CS	114	T6	T3–T8	–	500	3.58
22/M	15.0	CK	24	T11	T9–L2	–	500	4.0
23/M	13.0	GK	24	T4	**T1–T7**	–	1600	5.5
24/M	15.0	GK	24	T11	T8–L2	–	3500	6.0
25/F	6.5	CS	24	T11 + T12	T8–L2	–	700	6.5
26/M	6.9	CK	48	T7 + T8	T4–T11	–	800	4.0
27/M	9.5	CS	24	T10	T7–L1	+	1200	5.0
Mean	12.3 (1.1 to 20.7)		72 (24 to 115)				1015 (120 to 3500)	4.7 (3.1 to 6.5)

*CK* congenital kyphosis, *CS* congenital scoliosis, *GK* global kyphosis

Perioperative data were recorded, as well as radiographic outcomes (Table 3) and SRS-24 scores preoperatively and at follow-up visits. HRQoL questionnaires were filled by the patient or parents depending on the age of the patient. Typically, children below 10 years of age were assisted by their parents/caregivers to fill out the questionnaires. Patients were examined before and after surgery for their lower limb neurological function, walking ability, sitting, and standing balance. Preoperatively full spinal magnetic resonance imaging (MRI) and CT were taken in all patients. Children with congenital deformities underwent preoperative renal and cardiac ultrasound.

**Table 3:** Radiographic outcomes in 27 VCR patients

Patient	Major Cobb angle				Maximal lateral Cobb angle				Maximal coronal Cobb angle				Coronal vertical axis (mm) d*x* + , sin -			T12-S1 lordosis		
	Pre-op	Post-op	Final follow-up	%	Pre-op	Post-op	Final follow-up	%	Pre-op	Post-op	Final follow-up	%	Pre-op	Post-op	Final follow-up	Pre-op	Post-op	Final follow-up
1	88	38	38	57	59	51	46	22	88	38	38	57	32	28	54	41	41	48
2	34	14	14	59	17	25	18	6	34	14	16	53	18	28	5	59	55	64
3	55	25	25	55	2	14	14	86	55	25	25	49	17	30	25	33	39	40
4	69	22	27	61	69	22	27	61	56	18	22	61	5	5	0	103	46	54
5	49	11	8	84	49	11	8	84	33	11	11	67	0	5	5	70	55	45
6	82	40	43	48	82	40	43	48	1	1	1	0	0	−9	0	54	35	48
7	64	16	14	78	64	16	14	78	34	5	0	100	0	−15	0	77	65	61
8	92	54	53	42	92	54	53	42	5	5	0	100	23	23	18	55	47	28
9	39	3	5	87	49	6	8	84	39	3	5	87	−12	0	5	36	22	28
10	59	14	14	76	41	19	23	44	59	14	14	76	−14	0	3	86	59	65
11	35	4	4	89	53	13	25	53	35	4	4	89	12	0	24	18	45	24
12	81	30	32	60	70	36	41	41	81	30	32	61	17	55	23	47	27	38
13	32	15	24	25	32	10	16	50	32	15	24	25	−23	−22	0	64	43	43
14	25	4		84	2	4		84	25	4		84	−68	−40		35	31	37
15	39	21	29	26	39	21	29	26	23	15	5	78	−10	0	0	46	38	35
16	19	9	9	53	29	14	17	41	19	9	9	53	12	−10	−11	42	45	44
17	32	3	6	81	56	37	43	23	32	3	6	81	20	20	12	44	36	45
18	14	7	4	71	40	16	14	65	14	7	4	71	0	0	0	57	34	39
19	92	38	45	51	92	38	45	51	17	9	8	53	58	65	19	88	57	61
20	87	62	63	28	87	62	63	13	36	13	19	47	9	6	16	69	61	71
21	52	19	20	62	27	40	32	78	52	19	20	62	−8	25	−20	50	49	53
22	67	10	20	70	67	10	20	70	18	2	5	72	0	0	0	70	35	35
23	99	43	43	57	99	43	43	57	14	2	2	86	2	0	0	75	45	50
24	89	34	34	62	89	34	34	62	28	0	0	100	5	2	3	64	35	40
25	87	35	35	60	87	35	35	60	64	35	35	45	5	3	3	30	25	30
26	72	27	27	63	72	27	27	63	0	0	0	0	16	0	0	31	64	52
27	68	27	40	41	68	27	40	41	68	27	40	41	5	0	0	53	47	37
Mean				60.3				53.6				62.9						
Min				25.0				5.6				0						
Max				88.6				88.6				100.0						

### Surgical technique

PVCR was performed as described by Lenke et al. [1]. The posterior elements of the spine and one-third of the posterior part of the rib at the area of VCR were exposed. The pedicle screws were inserted [9]. Multiaxial reduction screws were used at the apical concave side and just below the resection level and radiographs were obtained to confirm their position (CD Legacy or Solera 5.5/6.0, Medtronic Spinal and Biologics, Memphis, Tennessee, USA). 5 cm of the medial rib head on both sides was resected and an extra pleural plane around the thoracic spine was created, with preservation of the segmental vessels and nerve roots (Pedicle Subtraction Osteotomy (PSO) Tool Set; Medtronic Spinal and Biologics, Memphis, Tennessee). The vertebral body and the disk above and below were identified and then resected up to the concave side. The resection was completed with removal of the concave-side pedicle. The posterior vertebral body wall was removed (a posterior vertebral wall impactor (PSO Tool Set)). Epidural bleeding was controlled by bipolar cauterization and human thrombin with gelatin matrix (FloSeal; Baxter US, Deerfield, Illinois). Final correction was obtained with exchange of the final long rod along with in situ bending in the coronal, and in the sagittal plane.

**Fig. 1:**
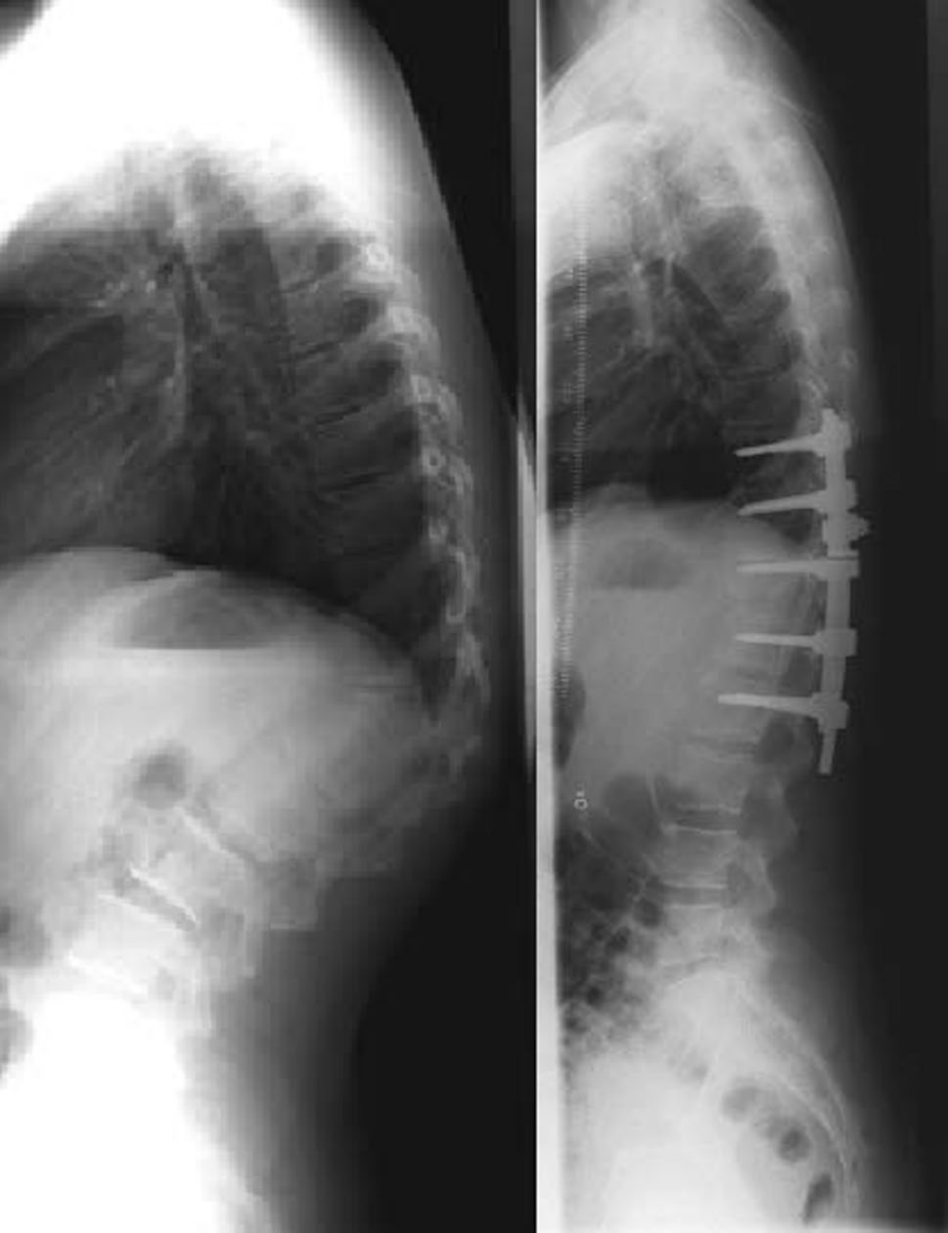
15-year-old boy with congenital kyphosis at the thoracolumbar junction. Standing lateral radiograph before and after Th12 PVCR and instrumentation from T9 to L2 at 2-year follow-up

**Fig. 2:**
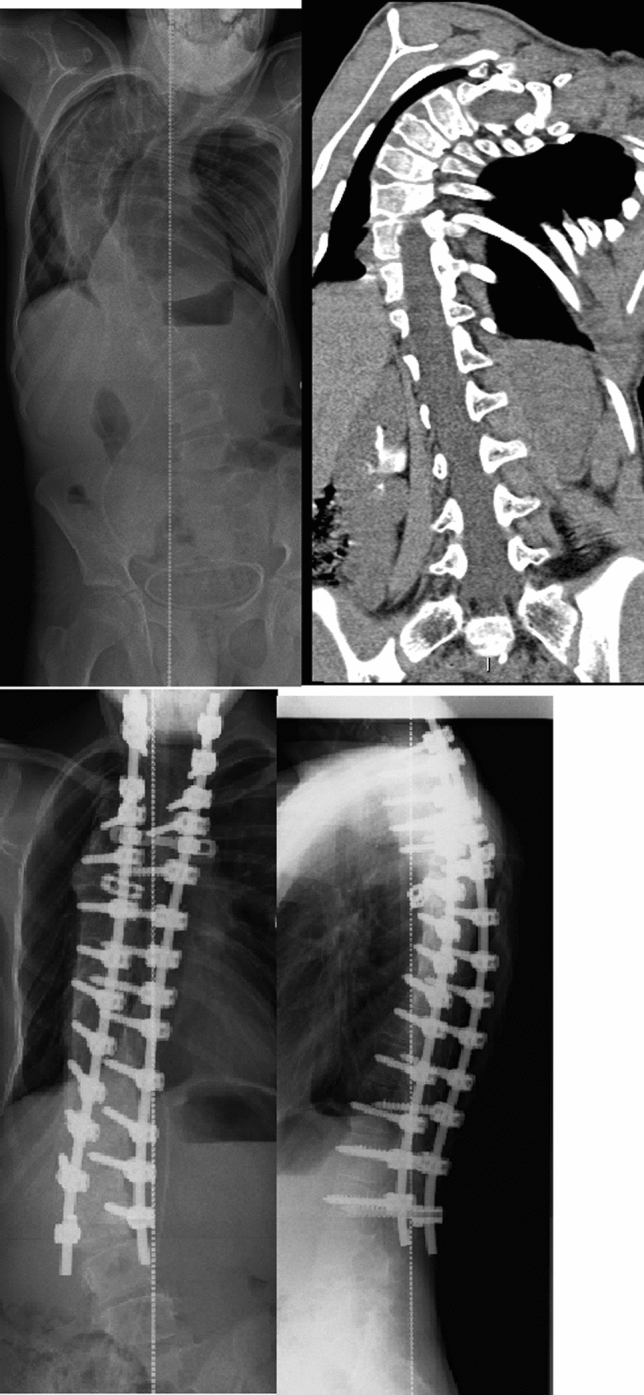
Severe AIS with preoperative halo traction. PVCR done to thoracic vertebra number 7 with cage postoperatively

If spinal cord monitoring demonstrated normal motor evoked potential (MEP) and sensory evoked potential (SEP) with shortening of the spinal column and the remaining defect after correction between endplates was < 5 mm, no cage was inserted and the gap was filled with cancellous bone chips (Fig. 1) A cage was used in cases where the defect after deformity correction remained more than 5 mm to expedite spinal fusion (Fig. 2) [5]. Five (18.5%) patients required a cage. Local bone graft taken from the wedge resection and the ribs was applied circumferentially.

### Perioperative management

Spinal cord monitoring was performed. Postoperatively patients were monitored in the pediatric intensive care unit and mean arterial pressure was maintained between 65 and 75 mmHg (24 h). All patients received intravenous prophylactic cefuroxime and vancomycin 30 min before incision; cefuroxime was continued for 72 h postoperatively. Thirteen patients (48.1%) were immobilized using a rigid thoracolumbosacral orthosis (TLSO) for 4 months.

### Radiographic parameters

The proximal thoracic, main thoracic, and thoracolumbar/lumbar curves were measured from anteroposterior radiographs and thoracic kyphosis (T5–T12), lumbar lordosis (T12–S1), and segmental kyphosis or lordosis were measured from the lateral radiographs using the Cobb technique [10, 11].

### SRS-24 questionnaire

SRS-24 -questionnaire [12] was filled out by the VCR patients preoperatively and at follow-up visits. The questionnaire has 7 domains: pain, general self-image, function from back condition, general level of activity, postoperative self-image, postoperative function, and satisfaction. A score < 4 in the SRS-24 pain domain (1 = severe pain and 5 = pain free) is considered clinically relevant [13].

### Healthy controls

Rather than the SRS-24, the healthy control groups filled out the SRS-22r, which is an improved and modified version of the original SRS-24 [12, 14]. Data for healthy control subjects were obtained from our previous study [15]. Two-hundred and seventy-two healthy controls were selected from a population register and were invited to complete and return the SRS-22r questionnaire [12, 14] between January 2012 and December 2015 [15]. Two healthy controls were matched for sex and age at final follow-up for each PVCR patient.

To compare PVCR patients with controls, we used the first 15 questions of the preoperative SRS-24 outcomes. Questions 1 through 15 of the SRS-24 correspond with questions 1, 2, 4, 5, 6, 8, 9, 11, 12, 14, 15, and 17 through 20 of the SRS-22r. These questions were used as the basis for 4 domains of comparison between preoperative SRS-24 scores for the PVCR treatment group and the healthy control groups: pain (SRS-24: 1, 2, 3, 6, 8, and 11; SRS-22r: 1, 2, 4, 8, 11, and 14), general self-image (SRS-24: 5, 14, and 15; SRS-22r: 6, 19, and 20), general function (SRS-24: 7, 12, and 13; SRS-22r: 9, 15, and 18), and general activity (SRS-24: 4, 9, and 10; SRS-22r: 5, 12, and 17) [12].

### Statistical analysis

Mean changes between baseline and 2 years were compared between the groups with linear mixed models for repeated measurements. Model included time as within factor and group as between factor, and also group x time interaction was included in the model. Assumptions were checked with studentized residuals. Results for VCR operated patients at 2 years after operation and control subjects were compared with Wilcoxon rank sum test. *p* values less than 0.05 (two-sided) were considered as statistically significant. The data analysis was generated using SAS software, Version 9.4 of the SAS System for Windows (SAS Institute Inc., Cary, NC, USA).

## Results

Twenty-seven patients underwent posterior vertebral column resection (Table 1). PVCR was performed as a primary surgery in 25 (92.6%) and as a revision surgery in two (7.4%) patients. Preoperative halo-gravity traction was used in two children (7.4%) (3 months). The mean intraoperative blood loss was 1015 ml (range 120–3500 ml) and operative time 4.7 h (range 3.1–6.5 h) (Table 2).

The mean coronal curve correction was 62.9% (range, 0–100%) and the mean lateral curve correction was 53.6% (range, 5.6–100%) at final follow-up.

### Complications

Complications were noticed in 12/27 patients (44%) in the VCR cohort. These included four cerebrospinal fluid leakages requiring suture of the leak and three pleural lesions requiring a chest drain. One patient developed pneumonia. One superficial wound infection was noticed. One patient had skin erosion over proximal thoracic pedicle screws (2 years after the PVCR) and needed revision surgery (unilateral removal of prominent screws and wound revision). One patient presented with one-sided lower instrumentation partial pedicle screw pull-out. The patient was asymptomatic and during the 2-year radiographic follow-up remained stable suggesting spinal union. Two patients developed junctional kyphosis and one of them needed revision and extension of instrumentation. Intraoperative neurological events (transient losses of intraoperative neurophysiologic monitoring) were observed in ten cases (37.0%), but these resolved in eight patients with additional circumferential decompression of the spinal cord and with reducing deformity correction intraoperatively. Postoperative neurological deficits were encountered in two patients (2/27, 7.4%). One patient had L5 nerve root deficit (ankle dorsiflexion) postoperatively, which resolved within 10 days. Another was iatrogenic medullar contusion and right-sided paraplegia postoperatively. This neural deficit resolved fully in 6 months (Table 3).

**Table 4:** Intra- and postoperative complications in the VCR cohort

	Complication	Time point	Outcome
Patient 1	Pleural lesion	At operation	Pleural tube postoperatively, resolved
Patient 2	Proximal junctional kyphosis	Noticed 3 months after operation	No symptoms, no revision needed
Patient 3	Superficial wound infection	< 1 week postoperatively	Resolved with antibiotic treatment
Patient 5	Dural lesion	At operation	No symptoms postoperatively
Patient 8	Pleural lesion, dural lesion, postoperatively pleural effusion, postoperative pneumonia	At operation, pneumonia during 1. postoperative week	From dural effusion no symptoms, pleural effusion resolved with pleural tube, pneumonia resolved with antibiotics
Patient 9	Iatrogenic medullar contusion	At operation	Total right-sided paraparesis, resolved fully in 6 months
Patient 12	Pleural lesion, pneumonia	At operation, pneumonia during 1. postoperative week	Pleural tube postoperatively, antibiotics, resolved
Patient 14	Dural lesion	At operation	No symptoms postoperatively
Patient 15	Dural lesion	At operation	No symptoms postoperatively
Patient 20	Skin erosion over the proximal thoracic pedicle screws	Two years postoperatively	Revision surgery: Removal of unilateral prominent screws and wound revision
Patient 24	Peripheral L5 nerve paresis	Noticed after operation	Resolved within days
Patient 25	One-sided partial, lower instrumentation pull-out	At 6 months FU	Spinal fusion developed during follow-up, no revision surgery
Patient 27	Proximal junctional kyphosis	At 1-year follow-up	Revision and extension of instrumentation 2 years after index surgery

### Health-related quality of life

In VCR group pain, self-image, and general activity improved during the 2-year follow-up, but the pain score was the only with statistically significant change (*p* = 0.0002). Compared to healthy controls, the VCR group had significantly lower total, self-image, and function scores (*p* < 0.05), but similar pain and activity scores at final follow-up (Table 5).

**Table 5:** Health-related quality of life (SRS domains) preoperative in VCR patients and at final follow-up in VCR group and in healthy controls

SRS domain	VCR patients preoperative mean (SD)	VCR patients at final follow-up	VCR patients change from preoperative *p* value	Healthy control group mean (SD)	VCR postoperative vs healthy controls *p* value
Pain	4.00 (0.67)	4.66 (0.73)	**0.0002**	4.73 (0.48)	0.65
Self-image	3.78 (0.75)	4.11 (0.96)	0.18	4.69 (0.48)	**0.0017**
Function	4.22 (0.43)	4.00 (0.79)	0.30	4.87 (0.31)	** < 0.0001**
Activity	4.60 (0.85)	4.69 (0.71)	0.08	4.71 (0.36)	0.88
Satisfaction	N/A	4.31 (0.81)	N/A	N/A	N/A
Total score	4.13 (0.44)	4.14 (0.69)	0.48	4.61 (0.40)	**0.0012**

Bold values indicate statistically significant

Preoperatively as well as at 2-year follow-up, one VCR patient reported moderate to severe pain during the last 6 months with use of question #1 in SRS questionnaire. However, five patients scored < 4 in the preoperative SRS-24 pain domain. The number of patients scoring < 4 was two at 2 years follow-up (*p* = 0.195).

Patients with surgical complications had similar SRS-24 total score as well SRS-24 domains at final follow-up. Age at surgery, type of deformity (scoliosis vs. kyphosis), number of fusion levels, primary vs. revision surgery did not affect SRS-24 total scores or majority of domain scores. However, self-image domain scores were better in younger age group (*p* = 0.04) and in scoliosis group (*p* = 0.04) preoperatively and postoperatively in younger age group (*p* = 0.02).

### Comparison with healthy controls

The mean pain domain scores and scores in general activity did not differ between the VCR patients and the healthy controls. The SRS pain domain score averaged 4.66 (2.33–5.00) in VCR group and 4.72 (2.83–5.00) in healthy control group (*p* = 0.65). The mean scores in function and self-image domains were significantly better in the healthy control group as compared with the VCR group (*p* < 0.05) (Table 4).

## Discussion

Posterior vertebral column resection provided satisfactory correction of angular and/or severe pediatric spine deformities. Children with PVCR showed improvement in HRQoL but remained at significantly lower level than age- and gender-matched healthy controls at 2-year follow-up. The overall risk of complications, 44%, is significant and although transient in all patients this information should be given to families.

### Strengths and limitations

The strength of this study is that VCR procedures were performed by a single surgeon using standardized technique and prospective data collection. The control group allowed comparison with healthy controls. HRQOL assessment was possible as only patients with normal intelligence were included. However, the study is limited by small sample size and retrospective design.

One limitation of this study was the somewhat different questionnaires used (the SRS-24 and SRS-22r); however, we chose to keep the same original SRS-24 questionnaire in the surgical treatment group to provide data from preoperatively to final follow-up postoperatively. To provide comparable questionnaires, we used the 15 most similar preoperative questions from the SRS-24 and SRS-22r, including 8 questions that were exactly the same, to form the pain, activity, self-image, and function domains. For the 7 questions that were not an exact match, the scoring was not identical, but close enough to make a valid comparison.

### Comparison with previous data

In our series, over 50% curve correction rate was achieved with 44% complication rate. Intraoperative neurological events were monitored in 37%, and in two patients also postoperatively, but during follow-up, these resolved fully. Suk et al. were the first to report results using PVCR in adult patients [5, 6]. In their series, complications occurred in 24/70 (34%) and 4/16 (25%) patients, including three (3/86, 3.5%) complete spinal cord injuries which were not transient. Lenke et al. reported PVCR for 35 consecutive pediatric spinal deformities, with no spinal cord-related complications and with 60% correction of the scoliosis [1]. Spiro et al. evaluated 10 children undergoing PVCR for congenital kyphosis with two patients requiring revision surgery for junctional kyphosis and with two intraoperative neuromonitoring changes without postoperative neural deficits [2]. In a multicenter cohort of 147 pediatric patients undergoing PVCR, Lenke et al. reported a 59% complication rate, as well as a 27% rate of intraoperative neurological events [16], none of the patients had permanent paraplegia. Hence, our results on curve correction and complication rate were comparable with previous reports.

Studies evaluating the HRQoL in VCR patients are sparse. Recently, Riley et al. evaluated HRQoL in a group of 54 patients (31 children) after minimum 5-year follow-up [8]. Of the 54 patients, 30 (55.6%) sustained complications and seven (13.0%) required a revision. Intraoperative neurological events occurred in 12.9% (16.1% pediatric), and 12.9% of pediatric patients had postoperative neurological deficits. Despite the high risk of complications, significant improvements were observed in pediatric group in the SRS self-image (0.9 points) and in the SRS satisfaction (1.8) at 5-year follow-up. They suggested that patients appreciate HRQoL improvement after the surgical procedure, despite high complication rates. Our findings showed significant improvement in the SRS-24 pain domain after PVCR. The minimal clinically important difference for SRS-22r pain domain scores in adolescents has been reported at 0.20 [17]. Data regarding the minimal clinically important values for the SRS-24 are lacking. In the present study, the mean pain domain score improved by 0.72 (95% CI, 0.38 to 1.06) in the PVCR patients. There was a relatively large standard deviation in the SRS-24 scores of the VCR group resulting into smaller statistical power to detect statistical difference between the study groups. At the end of follow-up, the SRS total score, self-image, function domains were at a lower level in the VCR group than in the healthy controls, but the pain and activity domains improved from preoperative to similar level than in the control group. Despite improvement in the radiographic parameters, the children undergoing PVCR had a significantly lower self-image score than the healthy controls.

The pediatric patients undergoing VCR for severe and angular spinal deformities rarely present with existing myelopathy and the indication to osteotomy is to prevent myelopathy and spinal cord dysfunction [18, 19]. Even if the patient receives a major improvement in the spinal deformity, it may not be quite clear to the families that without apical VCR there would remain a relatively high risk of future deterioration in the spinal cord. It remains unclear if the families understand the risks of this procedure and even though all complications in the current series were transient this information is important to be delivered into the decision-making process. The length of instrumentation and spinal fusion required to prevent further adding on or junctional issues are relatively long resulting into lower score of the SRS function domain.

## Conclusion

Health-related quality of life showed significant improvement in pain scores despite 44% risk of transient complications after PVCR. This HRQoL improvement remained at a significantly lower level than matched healthy control group.

## Data Availability

The data that support the findings of this study are available from the corresponding author upon reasonable request.

## Code availability

SAS software, Version 9.4 of the SAS System for Windows (SAS Institute Inc., Cary, NC, USA).
